# Up-Cycling of *Olea europaea* L. Ancient Cultivars Side Products: Study of a Combined Cosmetic–Food Supplement Treatment Based on Leaves and Olive Mill Wastewater Extracts

**DOI:** 10.3390/life13071509

**Published:** 2023-07-05

**Authors:** Raissa Buzzi, Irene Gugel, Stefania Costa, Sonia Molesini, Silvia Boreale, Erika Baldini, Nicola Marchetti, Silvia Vertuani, Patrizia Pinelli, Silvia Urciuoli, Anna Baldisserotto, Stefano Manfredini

**Affiliations:** 1 Department of Life Science and Biotechnology, University of Ferrara, Via L. Borsari 46, 44121 Ferrara, Italy; 2 Department of Chemical, Pharmaceutical and Agricultural Sciences, Via Fossato di Mortara 17, 44121 Ferrara, Italy; 3 Ambrosialab, Via Mortara 171, 44123 Ferrara, Italy; 4 Department of Statistics, Computer Sciences and Applications DiSIA—PHYTOLAB Laboratory, University of Florence, Via Ugo Schiff, 6, Sesto Fiorentino, 50019 Florence, Italy

**Keywords:** *Olea europaea*, agri-food industry by-products, up-cycling, hydroxytyrosol, cosmetic, food, cosmeceutical, nutraceutical

## Abstract

In recent years, a reversal of the global economic framework has been taking place: from the linear model, there has been a gradual transition to a circular model where by-products from the agri-food industry are taken and transformed into value products (upcycling) rather than being disposed of. Olive tree pruning represents an important biomass currently used for combustion; however, the leaf part of the olive tree is rich in phenolic substances, including hydroxytyrosol. Mill wastewater is also discarded, but it still contains high amounts of hydroxytyrosol. In this study, cosmetic and food supplement formulations were prepared using biophenols extracted from leaves and wastewater and were tested in a placebo-controlled study on healthy volunteers using a combined cosmetic and food supplement treatment. A significant improvement in skin health indicators (collagen density, elasticity, etc.) and a 17% improvement against Photo-induced Irritative Stimulus was observed.

## 1. Introduction

The current linear economic model, based on the typical axiom “extract, produce, use, and discard” [1], has become extremely disadvantageous. Therefore, in recent years, a reversal of the global economic picture has been taking place: the linear model is gradually evolving into a “circular model”. The Circular Economy consists of a system where waste and by-products are taken and transformed into value products (upcycling) rather than being definitively disposed of [2].

In this context, upcycling represents a valid strategy for the implementation of circular economy. Upcycling counters the notion that an object has no value once disposed of or must be destroyed before it can be re-entered into a new production cycle. We are thus referring to a sustainable use: the main idea is to re-evaluate the used material by suggesting new ways to re-use it [3]. In this regard, instead of using virgin raw materials, biomass could represent an interesting starting substrate for obtaining molecules of pharmaceutical, cosmeceutical, nutraceutical, or food interest [4,5].

Olive tree pruning represents an important biomass that can be profitably used for energy production by combustion; however, the olive tree leaves can be also considered a source of bioactive compounds [6].

In fact, they are rich in phenolic substances, including hydroxytyrosol, which can reach 2.28 mg per 100 g leaf extract and can be considered the most important phenolic component in the leaf district, associated with beneficial properties of olive leaves, particularly as an antioxidant, anti-carcinogenic and anti-inflammation agent [7].

Much work has already been carried out by the scientific community to investigate the potential benefits of polyphenols and a plethora of important biological activities have been attributed to olive “biophenols”. This term was coined to indicate bioactive phenols in olives instead of the larger term of polyphenols, which does not necessarily include active molecules [8].

**Figure 1:**
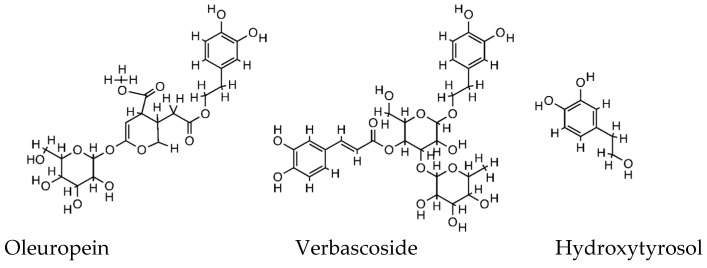
Structure of oleuropein, verbascoside, and hydroxytyrosol.

In nature, there are more than 100 different olive polyphenols, and although the extract composition differs between olive products (fruit, oil, leaves, and waste) and tissues (pulp and kernel), and it is greatly influenced by the extraction techniques, the major components include hydroxytyrosol and its derivatives, oleuropein and verbascoside (Figure 1). For these compounds, many pharmacological activities have been reported based on both preclinical (in vitro, ex vivo, and in vivo) and a few clinical studies [9].

In particular, antioxidant, anti-inflammatory, and antimicrobial properties, protection against UV damages, and the inhibition of dermal proteinases and anti-carcinogen properties have been demonstrated and suggest a high potential for the prevention and treatment of disease and the promotion of human health [9,10]. In addition, it is generally recognized that the consumption of these olive phytonutrients has certain health benefits, and adverse effects are not known so far [11].

The skin is a vital organ that protects the internal part of the human body from the external environment, maintains moisture and body temperature, and is part of the immune system [12]. As the outermost organ of the body, it is frequently and directly exposed to extrinsic agents acting as pro-oxidative stimuli [13]. These factors affect the rate of normal skin aging through the formation of free radicals, which attack the skin structures destroying the collagen and elastic fibers, impairing tension, elasticity, and hydration, with consequences such as dyschromia, changes in skin relief, spots, and wrinkles [14]. The areas of our body most exposed to the damaging exogenous factors, such as the face, are also the most visible in social life. Furthermore, high living standards have increased the number of individuals with both needs and desires for cosmetic amelioration [15]. The use of natural antioxidants as topical and/or systemic agents, which reduce the onset of oxidative stress, may help to protect and increase the efficacy of the skin’s biological system [16]. The combination of cosmetics (beauty from the outside) and food supplements (beauty from the inside) is a new multiple approach which provides a synergistic activity aimed to maintain the cells’ antioxidant power.

Polyphenols are among the most abundant antioxidants in the human diet and are present in different foods such as fruits, vegetables, cereals, and olive oil. Acting both as cosmeceuticals and nutricosmetics, they can be used in combined oral and topical administration [17]. A promising strategy for enhancing skin protection from oxidative stress is, in fact, to support the endogenous antioxidant system, both with the application of topical products and the intake of diet supplements, in order to complete the cycle “beauty from-inside-and-beauty-from-outside” [16].

Therefore, in view of this evidence, our study involved the design and development of innovative formulations based on olive biophenols deriving from waste materials, such as water and leaves, obtained through a patented extraction procedure [18]. Waste materials derived from ancient native *Olea europea* L. cultivars grown in the area of Castello di Monte Vibiano (PG), Italy. The unique geographic localization and high peculiarity of cultivars, well documented and preserved during the centuries by the local farmers, prompted us to start a research project devoted to the upcycling of such a particular by-product. The aim was to attempt a Cyclic Economy approach that can take advantage of the tradition and the peculiarities of such long-lived plants (more than 500 years old, in some cases).

In light of the above-mentioned considerations, cosmetics and food supplements formulations were prepared using the biophenols extract, and the anti-aging and protective effects of these formulations was assessed on healthy volunteers in terms of a combined cosmetic treatment applied alone and in association with an olive biophenols food supplement both based on the same extract.

## 2. Materials and Methods

### 2.1. Instruments

Derma Unit SSC 3 Hydration Probe (Corneometer^®^), DermaLab Combo Skinlab Elasticity Probe (Cortex—Hadsund, Denmark), DermaLab Combo Skinlab (Cortex—Denmark), solar simulator (Multiport UV Solar Simulator Model 601, 150 W), DermaLab Combo Skinlab Skin Color Probe (Cortex—Denmark), and Photochem^®^ system (Analytik Jena AG, Jena, Germany) were used. HPLC-DAD-MS HP-1260 liquid chromatograph Infinity II, equipped with a DAD detector and an LC/MSD API-electrospray (Agilent Technologies, Santa Clara, CA, USA), operated in negative and positive ionization mode. Mass spectrometer operating conditions were the following: gas temperature 350 °C at a flow rate of 10.0 L/min, nebulizer pressure 30 psi, quadrupole temperature 30 °C, and capillary voltage 3500 V. The fragmentor was set at 120 eV. Polyphenols were analyzed by using a column Lichrosorb RP18 250 × 4.60 mm i.d, 5 µm (Merck, Darmstadt, Germany). The eluents were H_2_O adjusted to pH 3.2 with HCOOH and CH_3_CN. A four-step linear solvent gradient was used, starting from 100% H_2_O up to 100% CH_3_CN, for 117 min at a flow rate of 0.8 mL/min. UV–VIS spectrophotometer «ONDA V-10» (Giorgio Bormac, Carpi, Italy) was used.

### 2.2. Investigational Products

The biophenols extract was supplied by MVB Italy s.r.l. Marsciano, Perugia, (Latitude: 42°55′15″ N Longitude: 12°21′02″ E Altitude above sea level: 187 mt.) and was obtained from leaves and Olive Mill Wastewater (*Olea europaea* L.) following the patented extraction process WO2005123603A1 (2005) [18], based on membrane filtration and final spray-dry in the presence of maltodextrins.

### 2.3. Quantification of Total Polyphenols

The determination of total phenols was performed using the properly modified Folin–Ciocalteu colorimetric method. Different concentrations of gallic acid were used as standards for the construction of the calibration curve (0, 50, 100, 150, 250, and 500 mg/L). A total of 20 µL of the extract was mixed with 1500 µL of the Folin–Ciocalteu reagent (diluted 1:15) and incubated at room temperature for 5 min; subsequently, 300 µL of a 20% solution of sodium carbonate was added. The mixture was then mixed with a vortex and incubated for 90 min at room temperature in the darkness. The absorbance was measured at 765 mm using a UV–VIS spectrophotometer. Purified water was used as the blank. The test was carried out in triplicate and the mean of three readings was used for the calculations of total phenolic content. The results were expressed as gallic acid equivalents (GAE): mg eq-gallic acid per 1 Kg of the extract.

### 2.4. Quantification of Total Flavonoids

The total flavonoid content was measured using the spectrophotometric method with a colorimetric assay [19] with minor modifications. A total of 2 mL of the extract solution was mixed with 3 mL of a 5% aluminum chloride solution. The samples were incubated for 30 min at room temperature and the absorbance of the reaction mixture was measured at 415 nm against a mixture solution as blank. The same protocol was performed for the standard solution of quercetin for the construction of the calibration curve. The test was carried out in triplicate and the mean of three readings was used for the calculations of total flavonoids. The total flavonoids content of the extract was expressed as mg quercetin equivalents (QE) per Kilogram of the sample (mg/Kg).

### 2.5. HPLC Analysis of the Main Polyphenols

**Figure 2:**
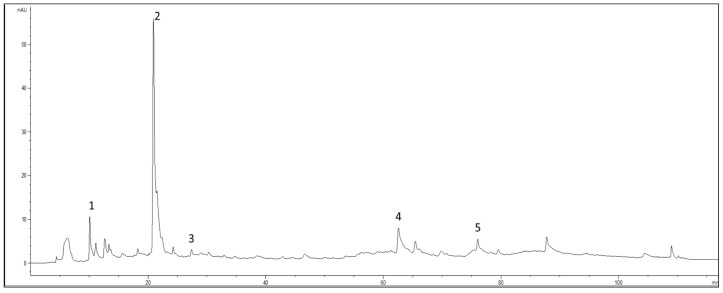
Chromatogram at 280 nm of the spray-dried extract with maltodextrins. *Peaks*: 1. Hydroxytyrosol glycol; 2. hydroxytyrosol; 3. tyrosol; 4. verbascoside; and 5. oleuropein.

A total of 99 mg of the extract was dissolved in 10 mL of distilled water acidified with formic acid at pH 3.2, and after complete solubilization the solution was centrifuged (14,000 rpm for 5 min). The supernatant solution was immediately used for the chromatographic analysis (injection volume 20 µL, column temperature 25 °C). The elution was performed using acidified H_2_O, pH 3.2, by HCOOH (Solvent A) and acetonitrile (Solvent B) at a flow rate of 0.8 mL/min, following the four-step linear gradient reported in Table 1. The chromatograms were acquired at 280 and 330 nm. The quantification of polyphenols was determined by using external calibration curves. In particular, hydroxytyrosol, hydroxytyrosol glycol, tyrosol, and oleuropein were quantified with HPLC-DAD at 280 nm, and verbascoside was quantified at 330 nm using five-point regression curves built with the available standards. Calibration curves with r^2^ ≥ 0.9998 were considered. The concentrations of the individual compounds were calculated by applying the appropriate corrections for changes in molecular weight. The chromatographic profile at 280 nm of the extract was reported in Figure 2.

### 2.6. Biophenols Extract Formulated Products

Using the extract, two different oil-in-water emulsions (O/W) were prepared and tested along with a food supplement provided by MVB.

*Emulsion A* had the following qualitative composition as INCI (International Nomenclature Cosmetic Ingredient): Aqua, Cetyl alcohol, Dicaprylyl ether, Myristyl myristate, Coco-caprylate, Cetyl palmitate, Ethylhexyl palmitate, Potassium cetyl phosphate, Tricaprylin, Cetearyl alcohol, Cetearyl glucoside, Phenoxyethanol, Glyceryl stearate, Oryza sativa bran oil, Sodium polyacrylate, Glycerin, Olea europaea leaf extract, Pentaerythrityl tetra-di-t-butyl hydroxyhydrocinnamate, Parfum, Ethylhexylglycerin, Olea europaea fruit extract, Olus oil, Sodium stearoyl lactylate, Tocopherol, Sodium lactate, Tocopheryl acetate, Sodium carboxymethyl betaglucan, Glycine soja sterols, Carnosine, and Lactic acid.

*Serum A* had the following qualitative composition as INCI: Aqua, Cetearyl alcohol, Olea europaea leaf extract, Cetearyl olivate, Coco-caprylate, Sorbitan olivate, Hydrogenated ethylhexyl olivate, Dicaprylyl ether, Elaeis guineensis fruit extract, Propanediol, Tocopherol, Maltodextrin, Ethylhexyl stearate, Squalane, Hydrogenated olive oil unsaponifiables, Benzyl alcohol, Xanthan gum, Parfum, Phytic acid, Ethylhexylglycerin, and Sodium hydroxide.

*Food Suppl. A.* Food Supplement, supplied by MVB Italy s.r.l., simply consisting of an aqueous solution of the above described biophenols extract.

### 2.7. Volunteers Selection, Consent, and Compliance with the Declaration of Helsinki

A single-center, open-label, efficacy study was designed. Forty-six healthy female volunteers were enrolled in this study. Informed consent was obtained from all subjects involved in the study.

- Inclusion criteria: Caucasian, age between 40 and 65 years old, female and absence of diseases immediately before or during the study;
- Exclusion criteria: subjects that did not fit the inclusion criteria, subjects with topical or systemic treatment with any drug in progress that may affect the outcome of the test (anti-inflammatories, steroids, etc.), pregnancy or breastfeeding, subjects affected by skin diseases in the areas selected for analysis, subjects affected by dermatitis, psoriasis or other dermatological diseases and subjects with known sensitivity to ingredients of the products;
- Drop-out: reasons for the premature exit from the study were the free choice of the subject and/or occurrence of adverse events.

The study took place at the Ambrosialab University of Ferrara Spinoff Company laboratories.

The trial protocol was conducted following an internal code of ethics applied by Ambrosialab that took into account the basic principles of the Helsinki Declaration. The tests provided by the study were conducted using non-invasive instrumental investigations, in accordance with the principles of Good Clinical Practice (GCP) and the Declaration of Helsinki (ethical principles for research involving human subjects). Informed consent was obtained from all subjects involved in the study. Not all the selected volunteers completed the treatment. In fact, for Group B, 4 subjects were lost: 2 due to health problems before the start of the study, and 2 during the follow-up stage due to loss of compliance with the study. All the other participants completed the treatment and attended all expected follow-ups.

### 2.8. Study Design

The selected volunteers were divided into 2 groups of 23 subjects each (Group A and Group B). The tests took place in an environment at a temperature of 21 ± 2 °C and with about 50% humidity. The volunteers were acclimatized 15 min before the measurements.

#### 2.8.1. Cosmetic Treatment

All participants of both groups had undergone cosmetic treatment, which consists of applying cosmetic products according to the following scheme:

- *Emulsion A*: an aliquot of emulsion (200 ± 20 mg) dispensed on random volar forearm every morning for 8 weeks;
- *Serum A*: an aliquot of serum (200 ± 20 mg) dispensed on the face and on the volar area of the treated forearm every evening for 8 weeks.

#### 2.8.2. Food Supplement

Only one of the two groups (Group B) was asked to take the food supplement *Food Suppl. A*, as follows: a 3 mL vial per day to be diluted in 500 mL of water or other drink to be drunk immediately or during the day for 8 weeks.

### 2.9. Dermal Assessment of Collagen Density, Skin Elasticity, and Hydration

To assess the treatment effects, measurements were performed at the start of the study (T0) and after 4 (T4) and 8 (T8) weeks. Measurements were also carried out on a skin area where no product was applied as a control.

The variations in cutaneous hydration of the external layer of the epidermis (stratum corneum) were assessed via the measurement of skin dielectric properties on the center of the forehead and the designated test site on both the treated and untreated volar forearm using a Derma Unit SSC 3 Hydration Probe (Corneometer^®^).

To measure the collagen density a DermaLab Combo Skinlab Elasticity Probe was used that could capture high-resolution images of the deeper layers of the skin. The technique was based on the measurement of the skin’s acoustic response when it undergoes the impact of an acoustic impulse at a known frequency. This acoustic impulse is partially reflected by the skin, and the rejected signal portion is collected by an ultrasonic transducer and processed to provide an image of the skin’s cross-section. Collagen measurements were performed on both the treated and untreated volar forearm and on the forehead.

The measurements of skin elasticity were performed with a DermaLab Combo Skinlab Elasticity Probe that was based on the application of suction on the skin surface. The suction method included an elevation phase and a retraction phase of the skin, which were controlled by infrared sensors in the probe chamber. One of the parameters related to skin elasticity was Retraction Time: a reduction in Retraction Time was an indication of an increase in skin elasticity. Measurements of elasticity were performed on both the treated and untreated volar forearms.

### 2.10. Dermal Assessment of Protective Efficacy 

As a result of oxidative stress, the release of free radicals causes an inflammatory response on the skin, which can be seen by an increase in skin redness. By measuring the degree of skin redness that an irritative stimulus produces on the skin before and after a treatment, it is possible to evaluate the ability of a treatment to enhance the natural skin defenses. Considering the different natures of radicalizing agents, two different analysis protocols have been developed for the evaluation of the irritative response to oxidative skin stress: one based on a chemical stimulus, and the other on a photo-induced one. In both cases, these methods are minimally invasive, in which a stimulus is applied to the skin of healthy volunteers that can determine a slight and transient irritative effect measurable in terms of erythema index.

#### 2.10.1. Test of Protective Efficacy against Chemically Induced Irritative Stimuli

In this test, the irritative stimulus consisted of a chemical agent that caused a temporary inflammatory response with a radical mechanism.

The chemical stimulus used to induce skin erythema consisted of applying a solution of methyl nicotinate (irritant and rubefacient agent) over a 1 cm^2^ area in the volar portion of both forearms. The contact time with the irritant agent was 3 min, after which it was removed and instrumental readings of the erythema index at 15, 30, and 60 min from the same application were taken using a DermaLab Combo Skinlab Skin Color Probe. The recorded values were the average of 4 measurements.

The protective effect of the treatment for chemically induced irritation was assessed by measuring the response of the skin (skin redness) at the start of the treatment (T0) and after 8 weeks (T8) both on the treated and untreated ventral forearm; a reduction in the degree of redness corresponded to a smaller irritative effect.

#### 2.10.2. Test of Protective Efficacy against UV-Induced Irritative Stimuli

The photo-induced stimulus consisted of a pre-established ultraviolet radiation dose calculated based on the minimal erythema dose (MED) performed using a xenon arc lamp solar light simulator (Multiport UV Simulator), according to specifications provided by the guideline ISO 24444:2010 for the in vivo determination of the sun protection factor. Thus, an incremental series of delayed erythema responses was induced on several small subsites on the skin and assessed for onset redness from 16 to 24 h after the UV radiation in line with the judgment of a competent evaluator.

The test was conducted by irradiating an area of 1 × 1 cm on both the treated and untreated ventral forearms with a dose of UV radiation corresponding to 1.5 times the MED value (1.5 MED). The instrumental measurements of the irradiated area were carried out before the irradiation and after 24 h by using the DermaLab Combo Skinlab Skin Color Probe. The protective effect of the treatment against UV-induced irritation was assessed by measuring the response of the skin (skin redness) at the start of the treatment (T0) and after 8 weeks (T8); a reduction in the degree of redness corresponded to a smaller irritative effect.

### 2.11. Antioxidant Capacity

All extracts, *Emulsion A*, *Serum A*, and *Food Supplement A*, were tested for their antioxidant capacity. For the analysis of the antioxidant activity of the cosmetic treatment, the photochemiluminescence (PCL) assay was used. This method, based on the methodology of Popov and Lewin [20], consisted of the photochemical generation of free radicals and their sensitive detection by chemiluminescence. This effect was achieved by optical excitation of a suitable photosensitizer (Luminol) which, when exposed to UV light, resulted in the generation of the superoxide radical O_2_^−^. The presence of free radical scavengers such as phenols resulted in the quenching of chemiluminescence that corresponded with a pronounced induction period [21].

The analyses, performed with a Photochem^®^ system (Analytik Jena AG, Jena, Germany), were conducted using a commercial ACL kit for lipid-soluble antioxidative substances. The cosmetic samples were dissolved in methanol and an amount of 20 µL of suitable solution was mixed with ready reagents according to the attached instructions. The prepared mixture was then analyzed in the Photochem^®^ device equipped with dedicated software for a quick recording of the results. Trolox, a water-soluble analogue of vitamin E, was used as a standard control and the results were expressed in µmol Trolox equivalents (TE) per g of sample. For each sample, the experiments were performed with three replicates. For the food supplement, antioxidant properties were investigated using the oxygen radical absorbance capacity (ORAC) method, and the scavenging activity against peroxyl radical was carried out based on a previously reported and modified protocol [22]. Sample solutions (mg/mL) and Trolox dilutions (40–240 µM) were prepared using phosphate-buffered solution (pH 7.4). In a 96-well black microplate (VWR), 25 µL of sample solution, Trolox dilution or phosphate-buffered solution (pH 7.4) used as a blank was placed in the wells. Measurements of fluorescence were carried out at 37 °C and recorded at 5 min intervals up 30 min after the addition of AAPH. The ORAC values, expressed as Trolox equivalents (µmol TE/g compound), were calculated according to the method of Cao et al. [23]. The antioxidant capacity of the tested compound was quantified via the integration and calculation of the area under the curve (AUC), relating it to that produced by the reference Trolox.

## 3. Results and Discussion

### 3.1. Quantification of Total Polyphenols and Flavonoids

Table 2 shows the values of total polyphenols and flavonoids in the extract.

The high concentrations of polyphenols and flavonoids demonstrate the efficiency of the extraction process and the potential of the resulting formulations in the nutraceutical and cosmeceutical fields.

### 3.2. Characterization and Quantification of Polyphenols in the Extract

The characterization of the main polyphenols in the extract (Table 3) showed the presence of high concentrations of hydroxytyrosol, a hydrolysate of oleuropein, which has been shown to have many useful nutraceutical and cosmeceutical properties. From the cosmeceutical point of view, there is evidence in the literature that hydroxytyrosol can act as an antiaging and anti-inflammatory molecule [24].

### 3.3. Antioxidant Capacity

As shown in Table 4, the PCL values for cosmetic formulations were considerably high in reference to other formulations previously investigated by us [25,26,27]. Similar observations can be made for ORAC values of the biophenols extract, and this also indicates good antioxidant activity, in particular when it is compared with other plant extracts studied by the same research group [28,29] or compared with recent literature data on food supplements [30].

Due to the different reaction mechanisms underlying common antioxidant assays, the antioxidant capacity of an extract added to preparations varying in ingredient composition depended on the method used. Therefore, in order to obtain a better estimate in terms of antioxidant capacity, it is appropriate to undertake the assay closest to the nature of the sample to be tested, especially when comparing products with different compositions such as dietary supplements and cosmetic formulations. On the basis of this consideration, it was decided to evaluate the antioxidant capacity of the food supplement with ORAC, the best-known food-grade antioxidant screening procedure, and of the cosmetic formulations with PCL, which has long been used by us as the best method for the determination of the antioxidant capacity in lipophilic matrices [25,26,27].

### 3.4. Dermal Assessment of Collagen Density, Skin Elasticity, and Hydration

#### 3.4.1. Skin Hydration

**Figure 3:**
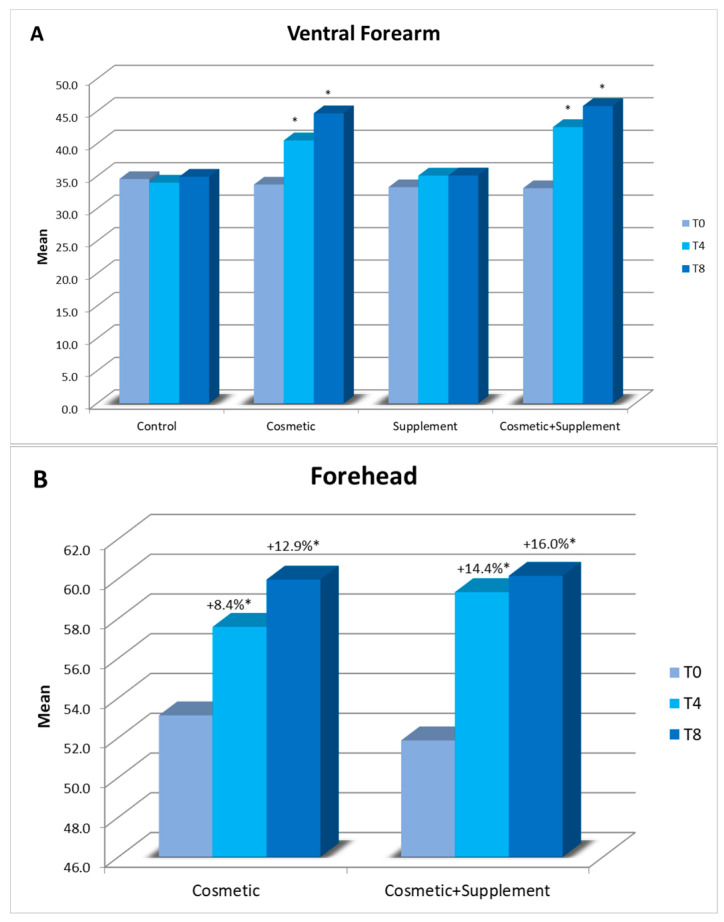
Skin hydration assessment using corneometer for ventral forearm (**A**) and forehead (**B**) at week 4 (T4) and week 8 (T8) vs. baseline. All the data are expressed in arbitrary corneometric units as an average of the recorded values. * *p* < 0.05.

A significant improvement on the ventral side of the forearm and face was noted in skin hydration, after 4 weeks of cosmetic products application, in comparison to baseline (Figure 3A,B), implying its efficacy in improving the hydration level of the skin. When the cosmetic treatment was combined with the supplement, no significant difference was noted in the ventral lower inner arm but a progressive and significant improvement was observed in face hydration, from week 4 onwards, until the end of the study. No changes were observed in the untreated control area.

#### 3.4.2. Skin Collagen Density

**Figure 4:**
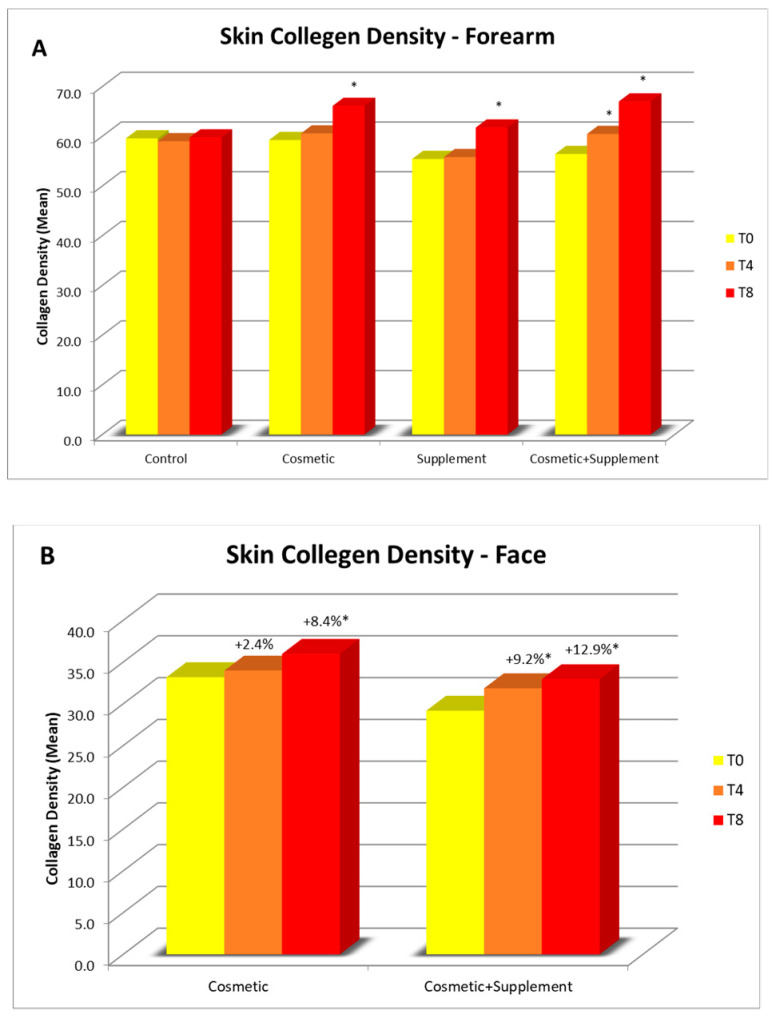
Skin collagen density assessment: (**A**) average collagen density values on the ventral forearms for all the treatments at week 4 (T4) and week 8 (T8) vs. baseline; (**B**) average collagen density values on the face at week 4 (T4) and week 8 (T8) vs. baseline. * *p* < 0.05.

A progressive improvement in skin collagen density was noted for all the treatments at all time points in comparison to baseline (Figure 4A,B). On the face, the increase recorded was 12.9% when compared with the group allocated to the cosmetic treatment alone (8.4%) (Figure 4B). No changes were observed in the untreated control area.

**Figure 5:**
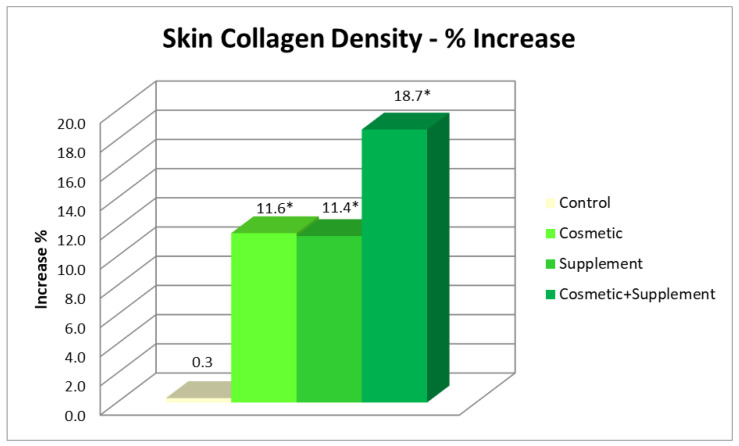
Percentage of increase in collagen density post 8 weeks’ treatment. * *p* < 0.05.

On both the ventral forearm and face after 4 weeks of study, a significant increase in collagen density was observed in the group allocated to the combined cosmetic–supplement treatment: on the forearm, an 18.7% increase was recorded in comparison to the area treated with the cosmetic alone (11.6%) and that treated with only the supplement (11.4%) (Figure 5).

**Figure 6:**
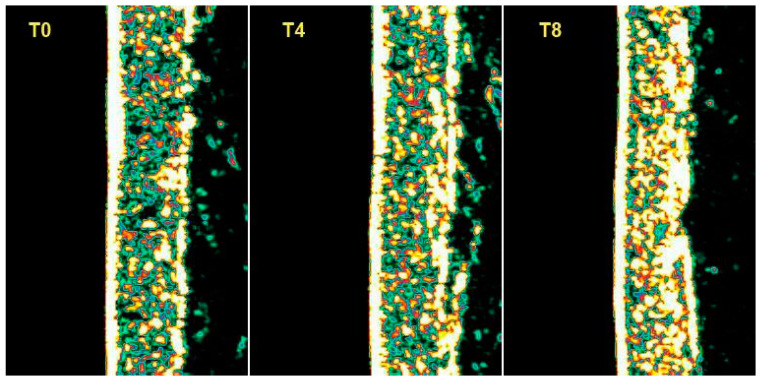
DermaLab combo skinlab variation in collagen density at selected times.

Figure 6 shows the collagen density increase during the combined treatment of cosmetic and supplement in one of the volunteers. Even after four weeks (T4), the collagen density was higher than in the control (T0); the treatment was even more effective after 8 weeks (T8); in fact the thickness of the collagen layer had clearly increased.

#### 3.4.3. Skin Elasticity

**Figure 7:**
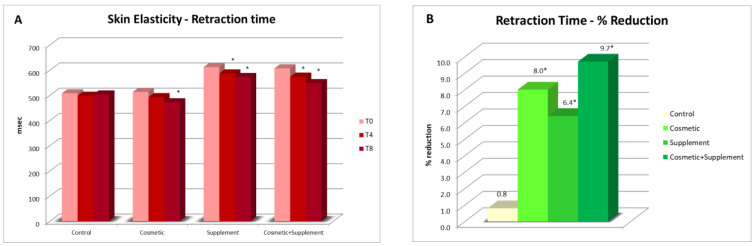
Skin elasticity assessment: (**A**) average skin elasticity values on the ventral forearms at week 4 (T4) and week 8 (T8) vs. baseline; the data were expressed as retraction time in milliseconds; * *p* < 0.05; (**B**) reduction percentage of retraction time post 8 weeks’ treatment.

For all the treatments, skin elasticity exhibited a slight but significant improvement over the 8 study weeks. In particular, in Group B (cosmetic–supplement treatment), a significant reduction in the retraction time could already be observed after 4 weeks on both the ventral treated forearm and the untreated one, implying that the supplement was efficacious in increasing skin elasticity. No significant change was observed in the control area not subjected to treatment (Figure 7).

### 3.5. Dermal Assessment of Protective Efficacy against Irritative Stimuli and Photo-Induced Stimuli

#### 3.5.1. Test of Protective Efficacy against Chemically Induced Irritative Stimuli

**Figure 8:**
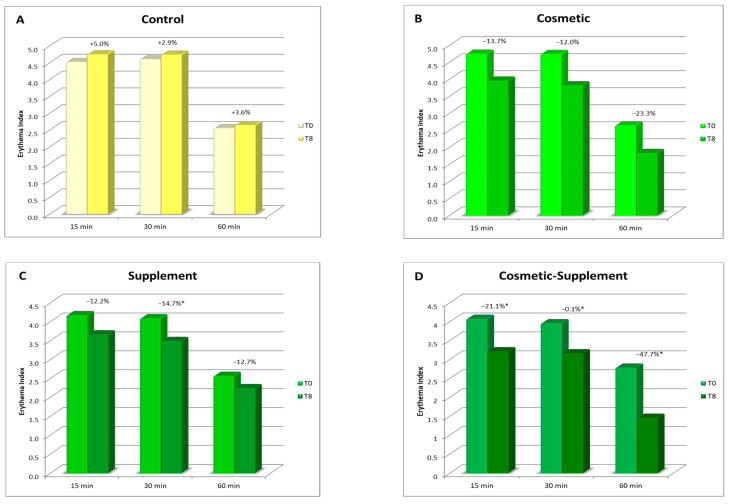
Erythema index assessment after chemically induced irritative stimulus: (**A**) average skin redness values on untreated ventral forearm at different intervals at week 8 (T8) vs. baseline; (**B**) average skin redness values on treated ventral forearm at different intervals at week 8 (T8) vs. baseline; (**C**) average skin redness values on untreated ventral forearm of Group B at different intervals at week 8 (T8) vs. baseline; (**D**) average skin redness values on treated ventral forearm of Group B at different intervals at week 8 (T8) vs. baseline. * *p* < 0.05.

A reduction in chemically induced skin redness was observed for all the treatments in comparison to the baseline. The most significant effect was noted at all the time points for the cosmetic–supplement treatment, with a redness reduction of more than 20% after 15 and 30 min of the application of the irritative stimulus and of almost 50% after 60 min. The effects of the cosmetic and supplement treatments alone were comparable, although not significant. No changes were observed in the untreated control area (Figure 8).

#### 3.5.2. Protective Efficacy against Photo-Induced Irritative Stimulus

**Figure 9:**
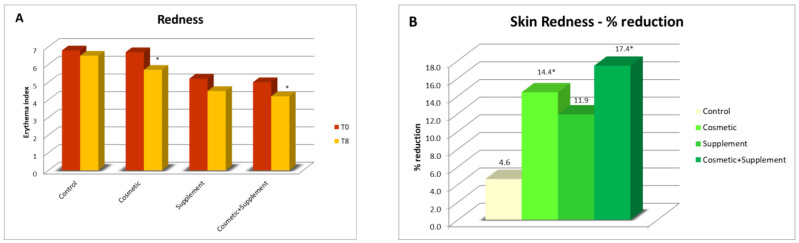
Erythema index assessment after photo-induced irritative stimulus: (**A**) average skin redness values for all the treatments at different intervals at week 8 (T8) vs. baseline; (**B**) reduction percentage of skin redness post 8 weeks’ treatment. * *p* < 0.05.

A reduction in photo-induced skin redness was observed for all the treatments in comparison to the baseline. In particular, when the treated forearms of Group A (cosmetic treatment) and Group B (cosmetic-supplement treatment) were compared, significant reductions of 14.4% and 17.4% were, respectively, observed. In the area subject to the effects of the supplement alone, the measured redness reduction was about 12% (not significant), while a negligible and completely random variation was observed in the control area not subjected to treatment (Figure 9).

## 4. Conclusions

A biophenols extract, obtained by an upcycling approach on waste materials from the olive agri-food industry, was investigated via the preparation and testing of cosmetic products and a food supplement. The combined action of the cosmetic (*Emulsion A*, *Serum A*) and supplement (*Food Suppl. A.*) applied, on 46 healthy volunteers, for a continuous period of 8 weeks, produced a significant progressive amelioration of the skin condition due to increases in collagen content, elasticity, and skin hydration. In addition, the treatment exerted a skin protective action, reducing irritative effects caused by chemical agents and UV radiation.

It was interesting to note that cosmetic treatment and supplement intake alone were also found to be effective in improving the skin parameters considered, although to a significantly lesser extent than the combined treatment. This further demonstrated the hypothesis that products acting at physiological and not pathological levels require a longer time and synergic action to achieve results as antiaging treatments. This work is of particular interest, being the first to demonstrate that it is possible to upcycle to high value ingredients wasted leaves resulting from the pruning of olive trees and mill wastewater, particularly when they are from very old cultivars, cultivated according to traditional methods, and are thus very precious biodiversity example (i.e., not from intensive cultivation). This represents yet-untapped wealth for Mediterranean regions that generally uses pruning byproducts as biomass for energy production and have to face costs for wastewater processing before discarding. Indeed, these extracts, containing a high amount of valuable hydroxytyrosol-based antioxidants, can be used in the preparation of cosmeceutical and nutraceutical ingredients. These latter, when applied in specifically designed products for synergistic treatments, gave significant improvements in skin health and appearance. Although only active at the physiological level, the combined treatment gives interesting improvements as compared to the single respective cosmetic and food supplements. In this regard, it can be affirmed that they are useful in inferring capabilities of prevention against skin damages due to early aging signs, in particular photo-aging.

The findings of this study demonstrate that the combined treatment had measurable effects that were consistent with the perceptions of the study participants. Overall, the participants rated the treatments as good or very good. However, one disadvantage of these products is the limited availability of raw materials. The raw materials used in the treatments, such as olive tree pruning waste and olive mill wastewater, can only be obtained during a specific period of the year.

Despite this limitation, the results of the study are highly encouraging, particularly in the context of a circular bioeconomy. The concept of a circular bioeconomy emphasizes the sustainable use of resources and the reduction of waste in the agri-food industry. By utilizing waste and by-products from the industry, such as those derived from unique cultivars, it is possible to develop other cosmetics and food supplements that contain high-value ingredients.

In the future, there is a potential for further development in this area. Research can focus on creating additional cosmetic and food supplement products using valuable components obtained from agricultural and food industry waste. This approach not only helps in reducing waste, but also promotes the utilization of resources that would otherwise go unused. The findings of this study provide a positive outlook for the future of circular bioeconomy practices in the agri-food industry.

## Figures and Tables

**Table 1:** HPLC gradient program.

Time (min)	Composition (%)	
	Solvent A	Solvent B
0.00	100	0
23.00	89	11
33.00	89	11
41.00	87	13
45.00	87	13
55.00	80	20
68.00	80	20
70.00	75	25
75.00	75	25
85.00	65	35
88.00	65	35
96.00	55	45
105.00	0	100
115.00	0	100
117.00	100	0

**Table 2:** The polyphenol content of biophenols spray-dried extract.

Total Content	[] mg/Kg
Flavonoids	10,435 ± 765
Polyphenols	57,791 ± 548

Total flavonoids expressed as QE mg/Kg and total polyphenols expressed as GAE mg/Kg.

**Table 3:** HPLC/DAD analysis of biophenols spray-dried extract.

HPLC Analysis	λ nm	[] mg/Kg
Hydroxytyrosol glycol	280	930
Hydroxytyrosol	280	7510
Tyrosol	280	330
Verbascoside	330	900
Oleuropein	280	790

**Table 4:** The ORAC and PCL values of biophenols spray-dried extract.

Product	PCL µmol TE/g	ORAC µmol TE/g
Food Suppl. A	------	901.51 ± 68.02
Biophenols extract	2994.15 ± 11.68	7515.98 ± 551.19
Emulsion A	95.43 ± 3.36	------
Serum A	310.20 ± 22.26	------

## Data Availability

The data presented in this study are available only in the article.
